# Initial experience with percutaneous coronary sinus catheter placement in minimally invasive cardiac surgery in an academic center

**DOI:** 10.1186/s12871-016-0203-4

**Published:** 2016-07-11

**Authors:** Satoshi Hanada, Hajime Sakamoto, Michael Swerczek, Kenichi Ueda

**Affiliations:** 1 Department of Anesthesia, University of Iowa Roy J. and Lucille A. Carver College of Medicine, 6JCP, University of Iowa Hospitals and Clinics, 200 Hawkins Drive, Iowa City, IA 52242 USA; 2 Department of Anesthesia, Akashi Medical Center, 743-33 Okubocho Yagi, Akashi, Hyogo Prefecture 674-0063 Japan

**Keywords:** Coronary sinus catheter, Minimally invasive cardiac surgery, Anesthesia, Retrograde cardioplegia

## Abstract

**Background:**

Placement of a percutaneous coronary sinus catheter (CSC) by an anesthesiologist for retrograde cardioplegia in minimally invasive cardiac surgery is relatively safe in experienced hands. However, the popularity of its placement remains limited to a small number of centers due to its perceived complexity and potential complications.

**Methods:**

We retrospectively reviewed all cardiac cases performed by one surgeon between December 2009 and April 2012. The reviewed cases were divided into two groups: cardiac cases with percutaneous CSC placement (CSC group) and cardiac cases without placement (control group). Anesthesia preparation time (APT) was then compared between the CSC group and control group. In the CSC group, cases were further divided into two groups. One group contained cases with an APT of less than 90 min (success group) and the other contained cases with an APT greater than or equal to 90 min or cases with CSC placement failure (delay/failure group). Patients’ characteristics, type of surgery, and transesophageal echocardiography (TEE) findings were compared between the two groups (success group vs. delay/failure group) to identify variables associated with prolongation of the APT or CSC placement failure.

**Results:**

Percutaneous CSC placement was required in 83 cases (CSC group). The catheter was successfully placed in 74 of those cases. We experienced one complication, coronary sinus injury after multiple attempts at placing the catheter. The mean APT was 102 ± 31 min in the CSC group (*n* = 81) and 42 ± 15 min in the control group (*n* = 285). We could not identify any variables associated with prolongation of the APT or catheter placement failure.

**Conclusions:**

The success rate of the placement was 89.1 % in our academic center. On average, placing the CSC added approximately one additional hour to the APT. This time is not an accurate representation of true catheter placement time, as it included time for preparation of the CSC, TEE, and fluoroscopy. We experienced one documented complication (coronary sinus injury), which was immediately diagnosed by TEE and fluoroscopy in the operating room. No variables associated with prolongation of APT or CSC placement failure were identified.

**Electronic supplementary material:**

The online version of this article (doi:10.1186/s12871-016-0203-4) contains supplementary material, which is available to authorized users.

## Background

In recent years, advancements in laparoscopic and thoracoscopic surgical techniques have created new ways to minimize incision size, facilitate a faster recovery, and, ultimately, reduce the length of hospital stay [1–3]. Applying this concept to cardiac surgery has resulted in the creation of minimally invasive cardiac surgery (MICS), which has surged in popularity over the last decade [4, 5]. MICS is performed with small incisions without full sternotomy and requires special skills for both the surgeons and anesthesiologists [6]. Retrograde cardioplegia can be administered percutaneously when placement of a traditional coronary sinus catheter (CSC) on the surgical field is not feasible during MICS. Anesthesiologists play an important role in the placement of a percutaneous CSC via the right internal jugular vein. The catheter placement is relatively safe in experienced hands; however, the popularity of its placement among anesthesiologists remains limited to a small number of centers due to its perceived complexity and potential complications [7, 8].

In December 2009, we started preforming percutaneous CSC placement during MICS. At that time, none of our anesthesiologists were experienced in the placement of a percutaneous CSC. When the decision was made to implement the procedure, two cardiac anesthesiologists at our institution were trained with one introductory lecture and one demonstration of CSC placement at an experienced facility. These two anesthesiologists at our institution then placed a percutaneous CSC with the help of a vendor’s technical representative. After performing the procedure several more times, these two anesthesiologists trained other cardiac anesthesiologists at our institution. Currently, seven cardiac anesthesiologists at our institution are able to perform the procedure.

In this article, we report our initial experience with the percutaneous CSC placement during MICS at our academic center. We retrospectively reviewed the success rate of the catheter placement, its complications, and the time required for catheter placement. We then analyzed factors that may prevent placement of the catheter or lengthen the placement time. In addition, we also reviewed the trend of the placement time over the study period to learn whether there was a learning curve associated with the procedure time.

## Methods

**Fig. 1:**
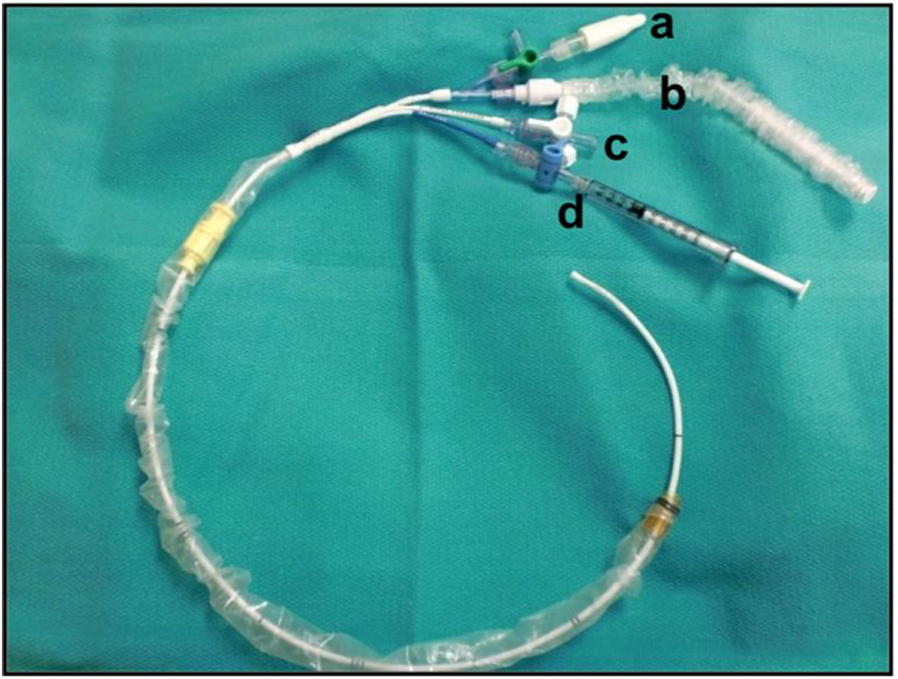
Percutaneous Coronary Sinus Catheter (Endoplege; Edwards Lifesciences, Irvine, CA). **a** Retrograde cardioplegia infusion port, **b** Stylet, **c** Coronary sinus pressure line, **d** Balloon infusion port

MICS, which required placement of the percutaneous CSC (EndoPlege; Edwards Lifesciences, Irvine, CA) (Fig. 1), was performed by one attending cardiac surgeon at our institution between December 2009 and April 2012. We conducted a retrospective review for all cardiac surgical cases (open heart surgery and MICS) performed by this surgeon during the above period. This study was approved by the University of Iowa Institutional Review Board. The reviewed cardiac cases were divided into two groups: the cardiac cases with the percutaneous CSC placement (CSC group) and the cardiac cases without the percutaneous CSC placement (control group). Anesthesia preparation time (APT), defined as the duration between anesthesia induction and the time the patient was ready for the surgical team, was then compared between the CSC group and the control group. The APT was obtained from our electronic medical record (Epic systems software). The difference between the two groups’ mean APT represents the mean of the additional time required for the percutaneous CSC placement.

In the CSC group, the reviewed cases were further divided into two groups. One group contained the cases with an APT of less than 90 min (success group), and the other contained the cases with an APT greater than or equal to 90 min or the cases with the CSC placement failure (delay/failure group).

The patients’ demographics (age, body mass index [BMI], sex), type of surgery, and transesophageal echocardiography (TEE) findings were compared between the two groups (success group vs. delay/failure group) to analyze any factors that may have delayed or caused failure of the CSC placement. The TEE findings that were reviewed included the presence of right atrium dilation, left atrium dilation, right ventricle dilation, left ventricle dilation, ascending aorta dilation, and presence of pacing lead(s). Univariate analysis was conducted to identify variables associated with delayed or failure of CSC placement. Continuous variables were compared with *t*-test or Mann-Whitney *U* test if appropriate. Chi square test was used for dichotomous variables. All analyses were performed with SPSS version 23 and a *P-*value <0.05 was considered statistically significant.

Lastly, the reviewed cases in the CSC group were divided into quarters chronologically by procedure date. We then compared the mean APT of each quarter to determine the learning curve of CSC placement skill. In addition, the trend of APT was evaluated by each operator (cardiac anesthesiologist) who performed CSC placement more than 10 cases over the study period.

## Results

**Fig. 2:**
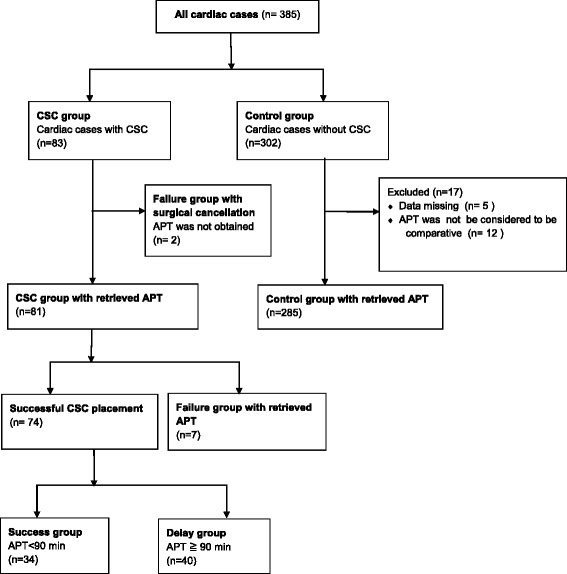
Flow Diagram of the case allocation

We retrospectively reviewed 385 open heart surgical cases performed by the attending cardiac surgeon between December 2009 and April 2012 (Fig. 2). Percutaneous CSC placement by the anesthesia team was required in 83 (CSC group) of the 385 cases. The catheter was successfully placed in 74 of those cases, for a success rate of 89.1 %. The percutaneous CSC placement was unsuccessful due to technical difficulty in 9 cases (failure group). The surgery was cancelled after anesthesia induction in two cases in the failure group. One was cancelled due to the complication of CSC placement (coronary sinus injury) after multiple attempts at placing the catheter. The other was cancelled after CSC placement failure because the surgery was not feasible to perform without a CSC. In these two cancelled cases, the anesthesia preparation time (APT) was not able be obtained because the ending time for APT (time for being ready for surgical team) was not recorded in the anesthesia chart.

CSC placement was not required in 302 cases (control group). Seventeen out of 302 cases were excluded from the data collection. In 5 cases, APT could not be obtained because the time was missing in the anesthesia charts. In the remaining 12 cases, patients were already intubated and/or had a pulmonary artery catheter placed prior to surgery in the Surgical ICU. These 12 cases were excluded from analysis because the APTs were not able to be considered compatible to the APTs of other cardiac cases, which required intubation and pulmonary catheter placement during the APT.

**Fig. 3:**
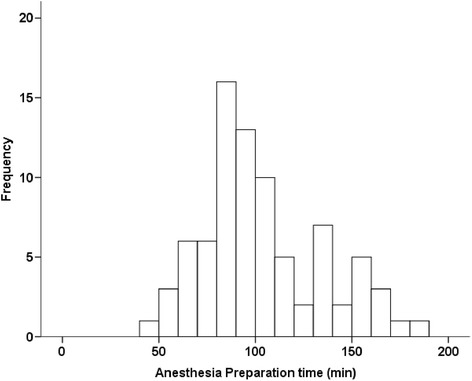
Histogram of Anesthesia Preparation Time (APT) in the CSC group (*n*=81)

The mean APT was 102 ± 31 min in the CSC group with retrieved APT (*n* = 81) (Fig. 3), and the mean APT was 42 ± 15 min in the control group with retrieved APT (*n* = 285) (Table 1). The mean difference was 60 min.

**Table 1:** Mean Anesthesia Preparation Time (APT*) between CSC Group and Control Group

	CSC group with retrieved APT (*n* = 81)	Control group with retrieved APT (*n* = 285)
APT (min)	102.3 ± 31.1	42.5 ± 15.3

*APT: duration between the time of anesthesia induction and the time the patient was ready for the surgical team

Upon univariate analysis, patient characteristics, type of surgery, and TEE findings were not significantly different between the two groups (success group vs. delayed/failure group) (Table 2).

**Table 2:** Univariate Results; Patient Characteristics, Type of Surgery, and TEE Findings

	Success Group; APT < 90 min (*n* = 34)	Delay Group; APT ≧ 90 min (*n* = 40) & Failure Group (*n* = 9)	*P*-value
Age (years)	65.6 ± 16.0	63.7 ± 13.4	0.46
BMI (kg/m^2^)	27.8 ± 4.0	28.0 ± 5.5	0.82
Male, Sex	23 (67.6 %)	31 (63.3 %)	0.68
Surgical type			0.73
MVR	13 (38.2 %)	23 (46.9 %)	
AVR	20 (58.8 %)	25 (51.0 %)	
Other	1 (2.9 %)	1 (2.0 %)	
LV Dilation	4 (11.8 %)	8 (16.3 %)	0.75
RV Dilation	4 (11.8 %)	8 (16.3 %)	0.75
LA Dilation	22 (64.7 %)	31 (63.3 %)	0.89
RA Dilation	6 (17.6 %)	14 (28.6 %)	0.25
AAo Dilation	3 (8.8 %)	3 (6.1 %)	0.69
Pacing leads	0 (0.0 %)	6 (12.2 %)	0.08

*MVR* mitral valve replacement/repair, *AVR* aortic valve replacement/repair, *LV* left ventricle, *RV* right ventricle, *LA* left atrium, *RA* right atrium, *AAo* ascending aorta

**Fig. 4:**
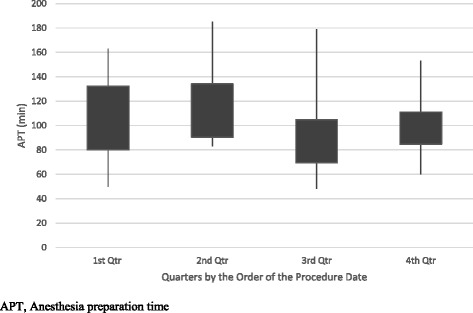
APT during the four quarters by the order of the procedure date in CSC group with retrieved APT (*n*=81)

**Fig. 5:**
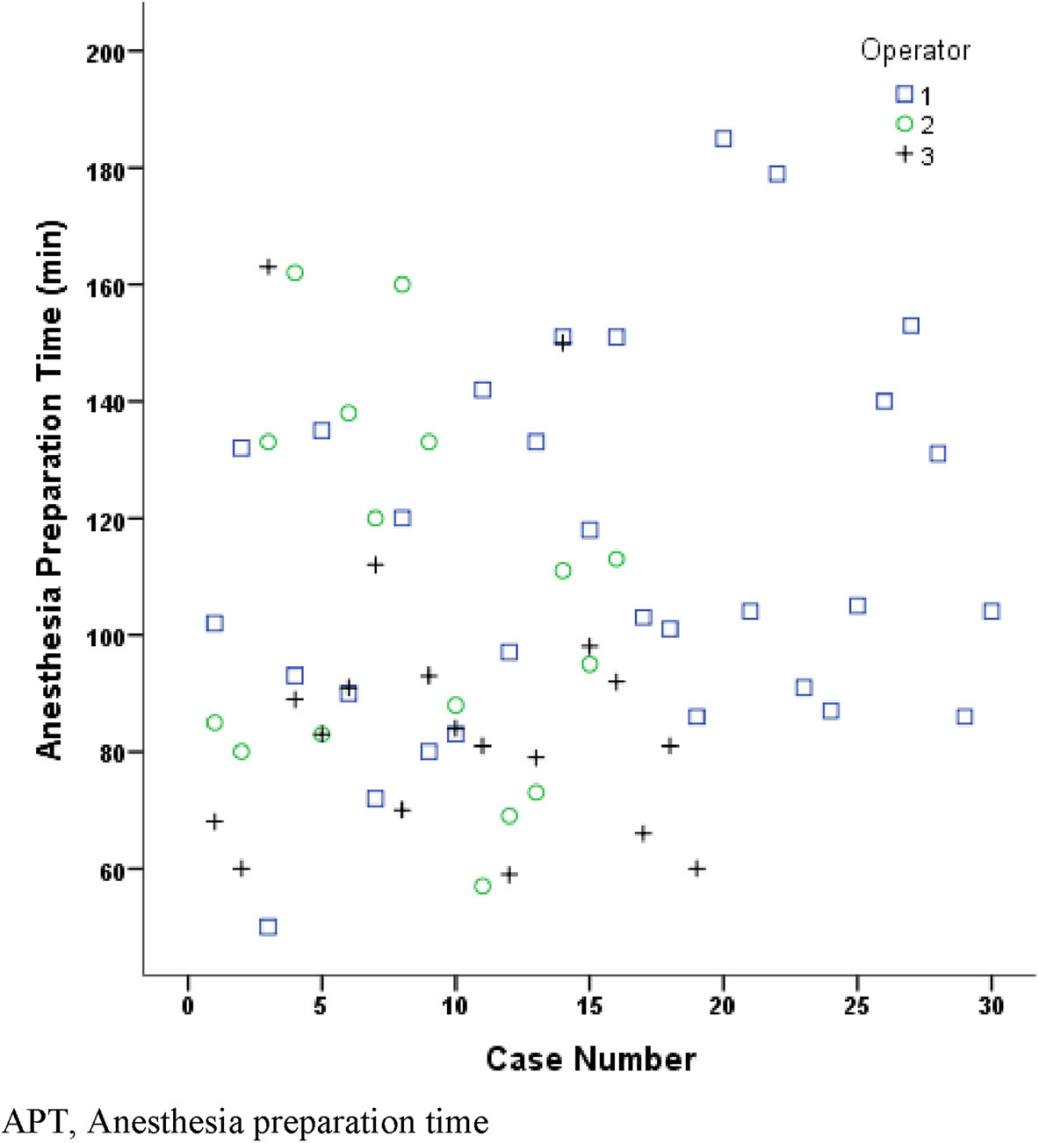
APT of each operator (operator 1, 2, and 3) by order of cases

In order to evaluate our learning curve for CSC placement, we divided the CSC group with retrieved APT (*n* = 81) into fourths chronologically based on the order of the procedure date. This allowed us to compare the mean APT of each quarter. The first 20 cases were in the 1^st^ quarter, the second 20 cases were in the 2^nd^ quarter, the third 20 cases were in the 3^rd^ quarter, and the last 21 cases were in the 4^th^ quarter, and the mean APT of each quarter was compared. There was no significant difference in the mean APT of each quarter (Fig. 4). Three operators (cardiac anesthesiologists) performed more than 10 cases over the study period. Operator 1 performed 32 cases (in which surgery was cancelled and APT could not be retrieved in 2 cases), operator 2 performed 16 cases and operator 3 performed 19 cases. There was no discernible trend of improvement in the APT for any operator (Fig. 5).

## Discussion

The placement of the percutaneous CSC can be challenging. Typically, the catheter engagement to the coronary sinus ostium is achieved under TEE guidance. Further advancement into the distal part of coronary sinus is performed under real time fluoroscopy [8, 9]. Familiarity with the catheter devices, TEE, fluoroscopy, and anatomy of the heart are necessary to place the catheter properly, safely, and in a timely manner.

The success rate of our initial experience for the catheter placement was 89.1 % (74/83) between December 2009 and April 2012. In surveying our cardiac anesthesiologists, we found that the catheter tip was engaged in the coronary sinus ostium under TEE guidance in a majority of the failed cases, but because of an acute curve in the coronary sinus or because of the small size of the coronary sinus, the catheter could not be advanced further despite multiple attempts using fluoroscopy. Langenberg et al. reported the success rate of initial catheter placement of 92 % (57 out of 62 cases) [10]. Lebon et al. reported a success rate of 94.7 % (90 out of 95 cases) [8]. The latest case series, from Labriola et al.*,* reported high success rate (98.6 %) using the next-generation percutaneous CSC (ProPlege; Edwards Lifesciences, Irvine, CA), which is equipped with a handle to change the curvature of the distal end of the catheter [11].

Although success rates of catheter placement in the cited cases were slightly higher than our results, catheter dislodgement occurred intra and postoperatively from 7–15 %. We did not have any recorded perioperative catheter dislodgment in our series. Dislodgment is a serious problem because repositioning the catheter is difficult while the surgery is in progress. Heart mobilization and/or positioning a venous cannula into the right atrium (RA) may cause dislodgment of the CSC. Placing the catheter into the distal portion of coronary sinus could prevent such intraoperative catheter dislodgement, although the optimal distance of catheter advancement into the coronary sinus is debatable. Shallow catheter placement may increase the risk of dislodgment during surgery, whereas deep catheter placement may compromise the optimal myocardial protection. Lebon et al. suggest “advancing the catheter as distally as possible, just before the great cardiac vein” [8]. Miller et al.*,* who experienced more than 500 cases, also agree to advance the catheter deeply within the coronary sinus [7].

**Fig. 6:**
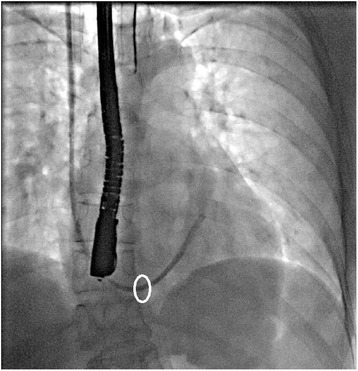
Confirmation of CSC position by contrast fluoroscopy. The usual catheter advancement is approximately 4 to 5 cm beyond the coronary sinus osmium. The white oval identifies the nominal projection of the coronary sinus ostium

In order to minimize the risk of catheter dislodgement, we advanced the catheter under live fluoroscopy guidance until the tip lay between 2/3 and 3/4 of the distance between the coronary sinus ostium and the left border of the heart. The usual advancement was approximately 4 to 5 cm beyond the coronary sinus osmium (Fig. 6). In addition, at our institution the balloon of the catheter tip was inflated during the entire surgery until the catheter was no longer needed, although there might be a potential risk of coronary sinus damage or thrombus formation with this strategy. Lastly, the catheter stylet was kept inside the cardioplegia lumen until the administration of cardioplegia was required. All of these three methods used at our institution could have reduced the risk of catheter dislodgment.

Various complications are possible with percutaneous CSC placement. Complications related to sheath introducer placement include pneumothorax and accidental arterial puncture [12, 13]. The occurrence of these complications is minimized when the introducer is placed under ultrasound guidance [14, 15]. Accordingly, we placed the introducer under ultrasound guidance routinely. Arrhythmias may frequently occur during the catheter manipulation. Plotkin et al. reported that two out of 11 patients developed transient atrial fibrillation during the CSC placement; one required cardioversion [16]. Arrhythmias often cease once the catheter manipulation halts; however, it may be wise to place external cardioverter–defibrillator patches on every patient before the catheter insertion in case prolonged arrhythmias occur. In our study, no prolonged arrhythmia events induced by the catheter placement were recorded.

Lebon et al. reported only one minor complication out of 96 cases; the complication was a local extravasation of contrast without evidence of coronary sinus rupture [8]. In contrast, Langenberg et al. reported that minor myocardial damage—such as hemopericardium, right ventricle (RV) hematoma, and contrast extravasation in the RV wall without hemodynamic instability—occurred in approximately 10 % of all cases [10]. In their study, the percutaneous CSC was placed in 62 patients who underwent cardiac artery bypass graft surgery with thoracotomy. After thoracotomy, the cardiac surgeon inspected the heart, palpated the coronary sinus region, and inspected the coronary sinus when possible. Langenberg et al. commented that “those instances of minor myocardial damage may not be clinically evident, but only observed after thoracotomy.” Considering their report, minor myocardial damage after the CSC placement may occur more often than we think, as the clinical signs of minor myocardial damage are not evident during MICS.

Serious complications such as injury to either the RA, RV or coronary sinus with pericardial tamponade are rare but possible. Abramson et al. reported a case in which a patient required full cardiopulmonary resuscitation followed by emergent sternotomy to repair the perforated RV after percutaneous CSC placement [17]. At our institution, we had one case of coronary sinus injury that occurred after we attempted catheter advancement into the distal coronary sinus. The coronary sinus injury was immediately diagnosed with TEE and fluoroscopy in the operating room [18]. The patient was hemodynamically stable and no intervention was required. However, the surgery was postponed for 3 weeks.

Catheter placement consumed considerable time at our institution. In a previous study, Lebon et al. reported a mean total procedure time of 16.1 ± 14.1 min [8]. The authors defined total procedure time as the time from the mobilization of the catheter in the RA to the time of confirmation of the final position by fluoroscopy during the procedure, which was measured by an assistant. We used the APT as a surrogate for the time required for the catheter placement. The APT was the time from anesthesia induction to the time the patient was ready for the surgical team. We retrieved the APT data from our anesthesia records. Subtracting the mean APT (42 min) in the control group (cardiac cases without the CSC placement) from the mean APT of the CSC group (102 min) gave the additional time required for the CSC placement, which was 60 min. On average, placing the CSC added approximately one additional hour to the APT. This is significantly longer than the time recorded in the Lebon et al. study; however, the retrieved APT time in our study is not an accurate representation of true catheter placement time because it includes time for CSC preparation, TEE, and fluoroscopy. TEE guidance is required for catheter placement, and a physician experienced in TEE needs to be in the operating room to assist with catheter placement. We often required two staff cardiac anesthesiologists to be in the room during the procedure: One acted as the operator and the other assisted with TEE. However, only one staff cardiac anesthesiologist was assigned to the case, and we occasionally needed to wait until another staff cardiac anesthesiologist was available to assist with the TEE. We also needed to wait until a radiology technician was available for fluoroscopy. Furthermore, in contrast with a private practice setting, teaching the assigned anesthesia trainee might also have contributed to the prolonged APT.

We divided the CSC group into two groups (success group and delay/failure group). The cases with an APT of less than 90 min were placed in the success group. Cases with an APT greater than or equal to 90 min and/or cases with CSC placement failure were placed in the delay/failure group. Ninety minutes was approximately twice the mean APT of the control group (42.50 ± 15.31 min), and thus we put cases requiring an APT of 90 min or more in the delay/failure group. Patients’ characteristics, type of surgery, and TEE findings were compared between the two groups to identify variable associated with prolongation of the APT or CSC placement failure. There were no significant differences between the two groups (Table 2). Existing pacing lead(s) can make catheter identification difficult on TEE and can also obstruct smooth catheter manipulation, which appeared to prolong the procedure or cause it to fail. In this retrospective study, all six cases with existing pacing lead(s) were in the delay/failure group. However, this finding was not statistically significant due to the study’s small sample size (*P* = 0.08). Lebon et al. reported that the CSC placement was difficult or impossible in patients with a hypoplastic coronary sinus or a prominent Thebesius valve [8]. In our retrospective study, however, the size of the coronary sinus, anatomical findings of the coronary sinus, and the presence of a prominent Thebesius valve were not able to be retrieved from medical records.

The 81 cases in the CSC group with retrieved APT were divided into quarters by the order of the procedure date, and the mean APT of each quarter was compared in order to assess the learning curve for CSC placement in an effort to shorten the procedure time. There was no discernible trend of improvement in APT between December 2009 and April 2012 (Fig. 3). Our institution tried to limit the number of cardiac anesthesiologists who performed the procedure to maximize the efficacy. In fact, the majority of cases (67 cases out of 83) were done by only 3 cardiac anesthesiologists. Operator 1 performed 32 cases, operator 2 performed 19 cases and operator 3 performed 16 cases over the study period. There was no correlation between APT and case number by each operator to determine the learning curve. A greater number of cases appear necessary to improve the efficiency of the procedure.

## Conclusions

With minimal training, it was possible to insert a percutaneous CSC successfully in nearly 90 % of patients undergoing MICS at our academic center. We experienced one documented complication out of 83 cases; the complication resulted in coronary sinus injury without catastrophic consequence. When CSC placement is attempted, the average time taken to get the patient ready for the surgical team is extended by approximately one hour. Substantial caseload may be necessary to gain experience in order to shorten APT of cardiac case with CSC placement.

## Abbreviations

APT, anesthesia preparation time; CS, coronary sinus; CSC, coronary sinus catheter; MICS, minimally invasive cardiac surgery; TEE, transesophageal echocardiography

## Additional file

Additional file 1: Available data. (XLS 95 kb)
